# MICRORNA REGULATORS OF THE SENESCENCE TRANSCRIPTOME

**DOI:** 10.1093/geroni/igz038.3076

**Published:** 2019-11-08

**Authors:** O’Wayne Rodney, Myriam Gorospe, Kotb Abdelmohsen

**Affiliations:** 1 Howard University, Washington, District of Columbia, United States; 2 National Institute on Aging, Baltimore, Maryland, United States

## Abstract

Cellular senescence is a state of indefinite growth arrest triggered in response to sublethal stresses such as telomere shortening, DNA damage, oxidative injury, oncogene activation, and hypoxia. Compared with proliferating cells, senescent cells are enlarged, display heterochromatic DNA foci, and express distinct subsets of proteins, including the enzyme β-galactosidase (β-gal). Previously, we identified transcriptome signature of senescent cells. We asked if these transcripts might be regulated by microRNAs (miRNAs). To address this question, we identified six miRNAs (miR-129-5p, -19a-3p, -128-3p, -124-3p, -340-5p, and -27b-3p) as potential regulators of subsets of transcripts differentially expressed during senescence. RT-qPCR analysis indicated that miR-129-5p, -19a-3p, -128-3p, -124-3p, and -340-5p were downregulated in senescent cells. We modulated these miRNAs in proliferating WI-38 fibroblasts and found that miRNA antagomirs did not show significant changes in β-gal activity. Interestingly, however, overexpression of miR-124-3p or miR-340-5p increased β-gal activity. We conclude that despite the decrease of miR-124-3p and miR-340-5p in senescent cells, their overexpression enhanced senescence as indicated by β-gal activity. Future analyses will focus on the mechanisms through which these miRNAs induce senescence and their physiologic and pathologic impacts in vivo.

